# Recent Molecular Insights into Agonist-specific Binding to the Mu-Opioid Receptor

**DOI:** 10.3389/fmolb.2022.900547

**Published:** 2022-06-13

**Authors:** Ferenc Zádor, Kornél Király, Nariman Essmat, Mahmoud Al-Khrasani

**Affiliations:** ^1^ Department of Pharmacology and Pharmacotherapy, Faculty of Medicine, Semmelweis University, Budapest, Hungary

**Keywords:** μ-opioid receptor, agonist-specific receptor activation, prototypic μ-opioid receptor agonist, TRV-130, PZM21

## Abstract

Opioid agonists produce their analgesic effects primarily by acting at the µ-opioid receptor (µOR). µOR agonists with different efficacies exert diverse molecular changes in the µOR which dictate the faith of the receptor’s signaling pathway and possibly it’s the degree of desensitization. Since the development of the active conformations of the µOR, growing data have been published in relation to ligand-specific changes in µOR activation. In this regard, this review summarizes recent data regarding the most studied opioid agonists in *in silico* µOR activation, including how these ligands are recognized by the µOR, how their binding signal is transmitted toward the intracellular parts of the µOR, and finally, what type of large-scale movements do these changes trigger in the µOR’s domains.

## Introduction

**FIGURE 1:**
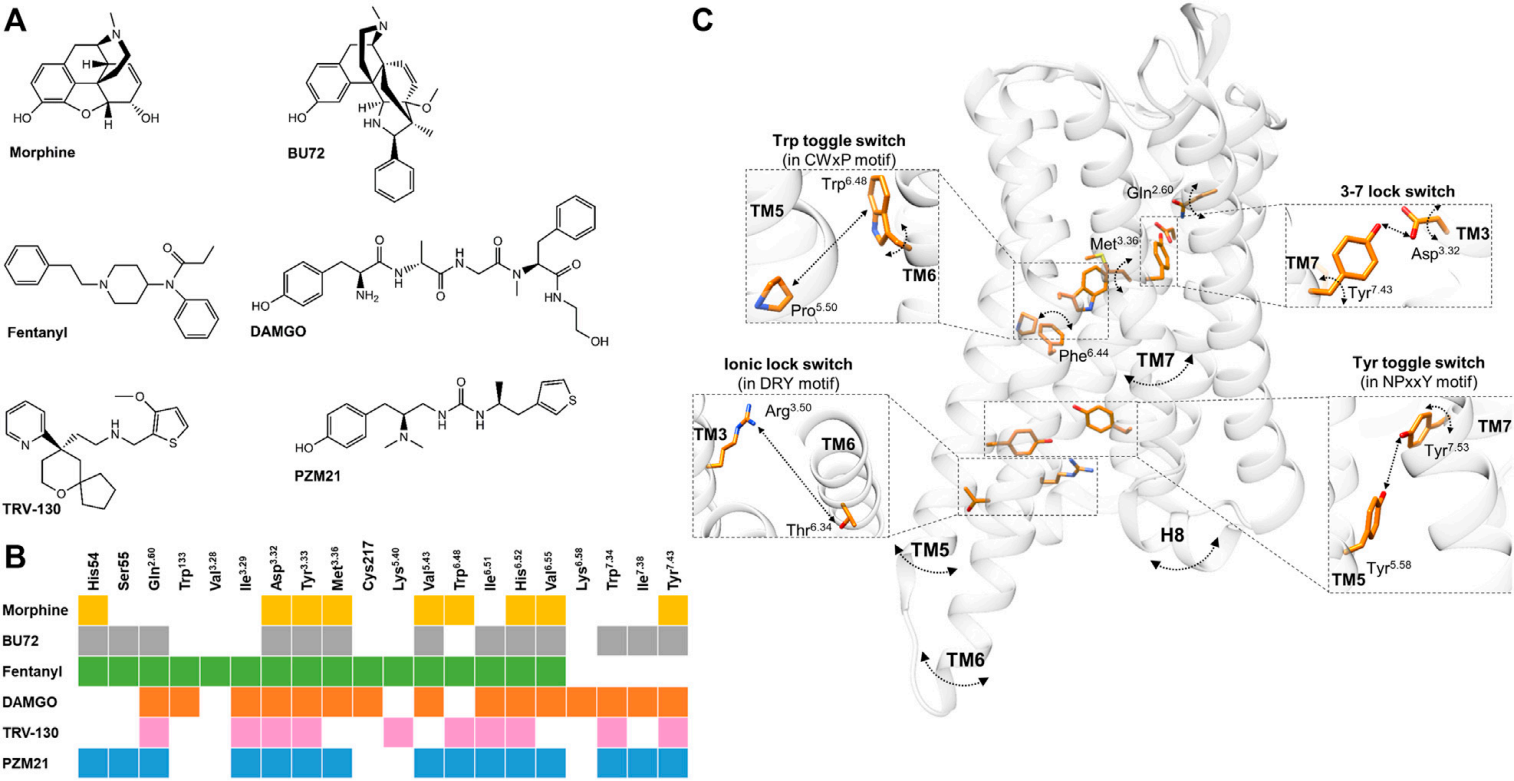
**(A)** Chemical structures of µOR-selective agonists discussed in the review. **(B)** The known µOR residual contacts of the indicated agonists. The original concept of the figure was based on Figure 4 of Podlewska and co-workers’ study (Podlewska et al., 2020) and extended by other data (Huang et al., 2015; Cheng et al., 2018; Koehl et al., 2018; Mafi et al., 2020; Ricarte et al., 2021). **(C)** Individual movements of the highlighted residues, molecular switches, and TM domains based on the data reviewed in the 3rd and 4th sections.. Participating residues are indicated in orange, arched arrows indicate the presence of spatial movements (but not the direction itself), while straight arrows depict the presence of altered distance between two residues. The corresponding agonists inducing these movements and alterations are not indicated for clarity; for details see in the 3rd and 4th sections. µOR is transparent for better visibility. The figure was constructed with UCSF Chimera 1.13.1 (Pettersen et al., 2004) based on Huang and co-workers using the BU72 co-crystallized active µOR structure (PDB: 5C1M) (Huang et al., 2015).

Growing data support that the rate of opioid side-effects including analgesic tolerance development strongly correlates with the pharmacodynamic properties of opioid ligands. Opioids with different efficacies distinctly induce molecular mechanisms related to tolerance, namely receptor phosphorylation and endocytosis, as the basis of G-protein coupled µ-opioid receptor (µOR) desensitization (Williams et al., 2013; Allouche et al., 2014; Lemel et al., 2020). It has been proposed that the selective and sequential phosphorylation of the C-terminus is due to the possible different conformational states of the receptor-triggered agonist specifically (Lemel et al., 2020). In recent years, we have gained more information regarding the nature of opioid agonists binding to the active conformation of the µOR (Huang et al., 2015; Koehl et al., 2018). This review will focus on the current knowledge of agonist specific residue contacts (Figure 1B), how the different agonists transmit the ligand-binding signal toward the intracellular receptor parts (Figure 1C), and finally, how these affect the orientation of certain receptor domains (e.g., transmembrane regions (TM) or intracellular loops (IL)) (Table 1), which eventually decide the faith of the receptor’s downstream signaling and the rate of desensitization. In addition, only data with the active conformation of the µOR will be reviewed here, namely the BU72 co-crystallized form and µOR-G_i_ complex co-crystallized with D-Ala^2^, N-MePhe^4^, and Gly-ol-enkephalin (DAMGO; PDB: 5C1M and PDB: 6DDF, respectively). Data on prototypic µOR-specific agonist ligands (Figure 1A), namely morphine, DAMGO, and fentanyl, will be reviewed alongside BU72, the first compound to be crystallized with the active conformational state of the µOR (Huang et al., 2015). TRV-130 and PZM21, newly developed G-protein-biased agonists, will be also reviewed (Figure 1A). In general, in the highlighted studies CHARMM (Brooks et al., 2009) and/or AMBER (Maier et al., 2015) force field was used, with 0.1–3.5 µs simulation time (in some cases, 24 µs; see Vo et al. (2020) in POPC (palmitoyl-oleoyl-phosphatidylcholine) lipid membrane model at ∼1 bar pressure and 310 K temperature in a ∼75–85 x 75–85 x 90–140 Å size simulation box. Some studies also used NMR spectroscopy to obtain dynamic structural information (Okude et al., 2015; Sounier et al., 2015). Such a pool of data will help us to better understand the basic molecular factors of ligand-specific receptor activation and tolerance, and allow us to purposefully develop opioids with delayed analgesic tolerance profiles and ameliorated side effects.

**TABLE 1:** Main differences and similarities within the highlighted ligands once bound to the µOR in terms of ligand recognition, binding signal transmission, and global movements.

Aspects	Differences	Similarities	References
Residue contacts, binding modes, and poses	Fentanyl has a deeper binding pose compared to morphine and has a unique His^6.52^ binding mode, which is dependent on the residue’s protonation state	All compounds interact with Asp^3.32^, Tyr^3.33^, and His^6.52^	Huang et al. (2015); Koehl et al. (2018); Lipiński et al. (2019); Dumitrascuta et al. (2020); Mafi et al. (2020); Podlewska et al. (2020); Vo et al. (2020); Lee et al. (2021); Mahinthichaichan et al. (2021)
	DAMGO binding pose extends further toward the ECLs	Fentanyl and morphine interact with TM7 to a similar extent	
	TRV-130 has stronger contacts with TM2 and TM3 compared to morphine and DAMGO	DAMGO and BU72 have similar binding poses	
	PZM21 has the strongest contact with Asp^3.32^ compared to fentanyl and morphine	Morphine, BU72, fentanyl, and DAMGO interact with Val^6.55^	
Ligand binding signal transmission	TM1 is necessary for morphine-induced µOR activation	Similar changes in microswitches with bound DAMGO and BU72	Huang et al. (2015); Schneider et al. (2016); Kapoor et al. (2017); Sader et al. (2018); Lipiński et al.(2019); Zhao et al. (2020); Liao et al. (2021); Ricarte et al. (2021)
	The H-bond within the 3–7 lock switch was stronger with fentanyl	Morphine and PZM21 have similar activated network paths toward the intracellular end of TM6	
	Different torsion angles of Phe^6.44^ and Trp^6.48^ with morphine and fentanyl		
	Overall, more information is transferred across the receptor when TRV-130 is bound compared to morphine		
	With PZM21 certain molecular switches behaved differently and the activated network paths were different at the end of TM7 compared to morphine		
	With PZM21, Trp^6.48^ and Tyr^7.43^ behaved differently compared to morphine or TRV-130		
Higher-order structural changes	With morphine, µOR exists in equilibrium between the closed and open conformations, with DAMGO the receptor mainly adopts the open conformation toward the intracellular space, while with TRV-130 µOR exists in equilibrium between the closed and open conformations, but with larger intracellular cavity	Morphine and fentanyl stabilize TM6 in active-like conformation from the activated state	Huang et al. (2015); Okude et al. (2015); Sounier et al. (2015); Kapoor et al. (2017); Mafi et al. (2020); Zhao et al. (2020); Liao et al. (2021); Ricarte et al. (2021)
	Fentanyl induces TM3 for a more upward conformation compared to morphine	Both BU72 and DAMGO induced ICL1 and H8 for a larger conformational change compared to TM5 and TM6	
	With BU72, TM6 makes a large outward movement and a smaller inward movement of TM5 and TM7		
	TM6 repositions when TRV-130 is bound, which hinders β-arrestin2 binding to phosphorylated µOR		
	With PZM21, intracellular ends of TM5–7 bent further outward compared to morphine, which is more favorable for G-protein binding		
	With PZM21, smaller ECL1–3 and ICL3 fluctuations compared to TRV-130		

## Ligand Recognition: Residue Contacts, Binding Modes, and Binding Poses

Based on site-directed mutagenesis and *in silico* studies, multiple conserved residues have been identified in the µOR binding pocket, which have significant roles in ligand orientation and receptor activation (Mansour et al., 1997; Manglik et al., 2012, 2015, 2016; Katritch et al., 2013; Kaserer et al., 2016; Koehl et al., 2018; Marino et al., 2018; Manglik, 2020; Ricarte et al., 2021). Hitherto, data on the agonist-specific residue contacts and binding modes will be reviewed in this section.

Despite morphine and fentanyl interacting with the same contact residues (Figure 1B), their binding poses were less overlapped (Lipiński et al., 2019). Accordingly, fentanyl is in close proximity to seven TM3 residues and three TM6 residues, while in the case of morphine these numbers are four and five with respect to the same transmembrane domains. They also interact with TM7 to a similar extent but with different positions. Fentanyl is also able to reach the ECL1, ECL2, and the N-terminus. These findings were later confirmed by another group (Ricarte et al., 2021).

Analyzing the dissociation of morphine from the µOR, it showed that morphine directly dissociated from the orthosteric site region and also transitioned to the vestibule region after the Asp^3.32^ salt bridge was disrupted (Ribeiro et al., 2020) (superscript numbering refers to the Ballesteros and Weinstein’s generic numbering scheme (Ballesteros and Weinstein, 1995)).

Fentanyl binds deeper compared to morphinan structures (for fentanyl it is indicated by a lower Δ*Z* value, the distance between the centers of mass (COM) of fentanyl and µOR *z* direction) and it can form a salt-bridge interaction between the piperidine amine and the conserved Asp^3.32^ (Vo et al., 2020) similar to DAMGO or BU72 (Huang et al., 2015; Weis and Kobilka, 2018). Vo and co-workers described a His^6.52^ binding mode unique to fentanyl, which was also dependent on the protonation state of this residue (Vo et al., 2020). Another study found that the dissociation pathways, time, the depth of insertion, and the strength of TM6 interaction of fentanyl are dependent on the protonation state of His^6.52^ (Mahinthichaichan et al., 2021).

In the case of BU72, most of its interactions with the active µOR are hydrophobic or aromatic. The phenolic hydroxyl group of BU72 interacts with His^6.52^ in a water-mediated fashion (Huang et al., 2015). There is also an ionic interaction between Asp^3.32^ and the morphinan tertiary amine structure of BU72. BU72 stabilizes the rearrangement of a triad of conserved residues upon receptor activation (Huang et al., 2015). BU72 also forms a hydrophobic surface with Ile^6.51^ and Val^6.55^ in TM6 and Ile^7.39^ in TM7, similarly to other morphinan structures (Figure 1B) (Huang et al., 2015). Another study demonstrated that BU72 binding poses distinct from the active µOR crystal structures and presumed that the high affinity and agonist character of BU72 is in part presented by its configurational entropy (Feinberg et al., 2017).

Koehl et al. found that the conformation of the active-state binding pocket and the orientation of the residues that interact with the agonist are highly similar between BU72 and DAMGO, despite the structural differences (Figure 1B) (Koehl et al., 2018). On the other hand, compared to BU72, the C-terminus of DAMGO extends further toward the ECLs. Another study with DAMGO has shown that the tyrosine of the peptide forms lipophilic contacts with Met^3.36^, Ile^6.51^, and Val^6.55^ residues and forms a charge interaction with Asp^3.32^ (Figure 1B) (Dumitrascuta et al., 2020).

It has been proved that TRV-130 has stronger interactions (a greater number of hydrophobic contacts) with TM2 and TM3 compared to morphine or DAMGO in β-arrestin2 stabilized with phosphorylated µOR (Mafi et al., 2020). Based on docking simulations, the protonated nitrogen ion of TRV130 formed electrostatic interactions with Asp^3.32^ and through its ring structure formed interactions with His^6.52^ (Figure 1B) (Cheng et al., 2018).

PZM21 interacts with the active µOR binding pocket by hydrogen bonds, hydrophobic interactions, and an ionic bond (Manglik et al., 2016). Podlewska and co-workers have compared PZM21 with fentanyl or morphine in docking and MD simulations in BU72 and DAMGO co-crystallized active structures (Podlewska et al., 2020). Interestingly, all compounds showed less stability in their orientations in the DAMGO co-crystallized conformation, especially morphine, meaning that their initial and final binding orientations were significantly different during the simulation. They also found that during simulation time, PZM21 had more contacts with Asp^3.32^ in both crystal structures compared to fentanyl or morphine (Podlewska et al., 2020). Another recent study compared PZM21 to morphine in MD simulations and found that besides PZM21 interacting with key residues Asp^3.32^ and Tyr^3.33^ of TM3 (Figure 1B), similar to morphine, yet it strongly interacts with Tyr^7.43^ of TM7 (Figure 1B), as indicated by a higher percentage of interaction fractions in H-bonds (Liao et al., 2021). Finally, Lee and co-workers have performed molecular docking with new potential biased µOR agonists, where they also compared these novel compounds to TRV-130 and PZM21 for control. Here, they found that TRV-130 and PZM21 failed to accomplish contact with Val^6.55^ in contrast to the novel compounds, which is heavily involved with hydrophobic interactions (Lee et al., 2021).

## Ligand Binding Signal Transmission

The subtle changes in the ligand-binding pocket induced by the bound ligand trigger further delicate changes through a channel of residues within certain TM domains. These changes transmit the ligand-binding signal from the ligand-binding site to the cytoplasmic region of the receptor (Weng et al., 2017; Liao et al., 2021). Some of these groups of residues are generally termed as molecular switches and they are conserved across the GPCR family. Among these, the 3–7 lock switch, the NPxxY motif (Asn-Pro-Xaa-Xaa-Tyr), the tyrosine (Tyr^7.53^) toggle switch, the Trp^6.48^ rotamer toggle switch, ionic lock (or DRY motif, Asp-Arg-Tyr), or the transmission switch (or CWxP motif, Cys-Trp-Xaa-Pro) have been described to be altered in an agonist specific manner in the µOR and will be discussed in this section, among other related data. The role of these molecular switches has been described in detail in other studies (Lagerström and Schiöth, 2008; Nygaard et al., 2009; Chabbert et al., 2012; Trzaskowski et al., 2012; Marino et al., 2018; Filipek, 2019) and due to length limitations will not be discussed here.

A study demonstrated that the conformations of certain residues (Met^3.36^ and Gln^2.60^) were different compared to morphine and fentanyl bound states (Figure 1C) (Ricarte et al., 2021). These differences affected the Asp^3.32^−Tyr^7.43^ H-bonding (3–7 lock switch) (Figure 1C), which was stronger when fentanyl was present (indicated by higher H-bond occupancy values) (Ricarte et al., 2021). They also found that the conformational changes in the NPxxY motif were consistently induced in the more stable active-like state by fentanyl (Ricarte et al., 2021). These specific changes might explain the higher efficacy of fentanyl. Another study proposed that the N-aniline ring of fentanyl mediates µOR β-arrestin coupling through the Met^3.36^ residue (de Waal et al., 2020). Additionally, a clear difference was shown in torsion angles of Trp^6.48^ between morphine and fentanyl (Figure 1C) (Lipiński et al., 2019). Also, the frequency changes of the torsion angles of Phe^6.44^ were considered the main difference between morphine and fentanyl. The same study revealed differences between morphine and fentanyl in the 3–7 lock switch and being tighter in the presence of morphine (Figure 1C) (Lipiński et al., 2019).

Sena et al. showed that morphine tends to drive the receptor toward increasing the distance in the 3–7 lock switch (Figure 1C) and found an important conformational change in TM5 when morphine was present (Sena et al., 2021). It is worth noting that MD simulations have been performed with morphine and a µOR splice variant lacking the complete TM1 (Majumdar et al., 2011,^2012^; Lu et al., 2015) where TM1 truncation results in the loss of key interactions that are necessary for morphine-induced µOR activation (Sader et al., 2018).

Huang and co-workers revealed an extensive network of polar interactions between the orthosteric binding pocket and the G-protein coupling interface, which rearranges upon receptor activation with BU72 (Huang et al., 2015). The NPxxY motif is also involved in this polar network and moves inward toward the TM5 upon activation (Figure 1C) (Huang et al., 2015). Later on, they found similar changes in the microswitches when DAMGO was bound to the µOR–G_i_ protein complex structure (Koehl et al., 2018).

Cheng and co-workers compared BU72 and TRV130, where the stability of Asp^3.32^ was lower with TRV-130 compared to BU72 (Figure 1C) since the dominant torsion angle was ∼ -12° and occupied ∼23% of the simulation time in the presence of TRV-130 (BU72: ∼28°, ∼45%) (Cheng et al., 2018). A study analyzed the allosteric communication between the orthosteric binding pocket and the intracellular region of the µOR with TRV-130 compared to morphine (Schneider et al., 2016). According to contact probability calculations, TRV-130 only communicated with residues of the intracellular end of TM3 and there was no strong contact with residues at the end of TM6. Morphine allosterically regulated significant interactions with the intracellular ends of both TM3 and TM6. Additionally, the network of side-chain interactions adjacent to TRV-130 was significantly smaller compared to morphine (Schneider et al., 2016). Also, when TRV-130 was bound, the residues in the EC2 and EC3 loops of the µOR formed a substantially extensive network of polar interactions when compared to morphine (Schneider et al., 2016). Kapoor and co-workers had found that more information is transferred across the receptor in TRV-130-bound µOR than in morphine-bound µOR based on transfer entropy analysis; for instance, the three extracellular loop regions are not involved entirely in any information transfer in the case of morphine (Kapoor et al., 2017).

Another study has found that morphine- and PZM21-activated network paths toward the intracellular end of TM6 were mostly identical, but the paths to the end of TM7 were evidently different (Liao et al., 2021). The same study also compared three key molecular switches, the ionic lock (DRY), transmission (CWxP), and Tyr toggle switches. Here, they found distance and rotational changes between morphine- and PMZ21-bound µOR, which affect the positions of TM5-7 (see later) (Figure 1C) (Liao et al., 2021). In another MD simulation, they compared TRV-130 and PZM21 with morphine, and one of the main differences was that the side chain of Trp^6.48^ (Figure 1C) was reversed with a delay with PZM21 compared to morphine (300 vs. 50 ns) and that Tyr^7.43^ side chain (Figure 1C) rotated with less fluctuation range compared to TRV-130-bound µOR (PZM21: 100°–175° vs. TRV-130: 100°–150°) (Zhao et al., 2020). These results also point to the low potency and lower bias effect of PZM21.

## Higher-Order Structural Changes, Global Movements

With GPCRs, the subtle changes in the ligand-binding pocket and ligand binding signal transmission throughout the TM domains add up to large, global toggle switch movements of the TM domains (Nygaard et al., 2009; Venkatakrishnan et al., 2013). These movements are crucial in the receptor inactive–active conformation transition (Huang et al., 2015; Zhou et al., 2019). However, regarding the µOR, there are multiple data pointing out that agonists with different efficacies or functional selectivities trigger these large movements differently or to a different degree. Such data will be reviewed in this section.

A study comparing fentanyl and morphine showed that fentanyl selects for more upward conformations of TM3 than morphine (+0.6 Å vs +0.2 Å) (Ricarte et al., 2021). Additionally, both compounds are able to stabilize an active-like conformation of TM6 in simulations initiated from the activated state; however, only fentanyl can achieve the same when starting from the inactive state of the receptor. This difference may contribute to the greater efficacy of fentanyl relative to morphine.

In the case of BU72, upon activation, TM6 makes a large 10 Å outward movement and smaller inward movement of TM5 and TM7 (Figure 1C) (Huang et al., 2015). Complementing these data in the presence of a G-protein mimetic nanobody in solution-state NMR, a weak allosteric coupling was revealed between the agonist-binding pocket and the G-protein-coupling interface (TM5 and TM6) (Sounier et al., 2015), similar to that observed for the β2-adrenergic receptor (Manglik et al., 2015). Most interestingly, in the presence of BU72 or DAMGO alone, ICL1 and H8 showed larger conformational changes (Figure 1C) (indicated by larger spectral signals) compared to TM5 and TM6, suggesting that these domains might play a role in the initial interaction with the G-protein (Sounier et al., 2015).

Okude et al. studied the NMR signals from methionine residues of the µOR in the morphine-, DAMGO-, and TRV-130-bound states. They found that when morphine was bound, µOR exists in equilibrium between the closed and open conformations; in the DAMGO-bound state, the receptor mainly adopts the open conformation. Upon TRV-130 binding, µOR exists in equilibrium between the closed and open conformations; however, in such cases, the open conformation adopts a larger intracellular cavity (Okude et al., 2015). The study also demonstrated that the population of each open conformation defines the G-protein- and arrestin-mediated signaling levels in each ligand-bound state.

Kapoor et al. found that morphine-bound μOR motions involved the cytoplasmic ends of only TM6, TM3, and TM5 (Figure 1C). On the other hand, the TRV-130-bound μOR motions involved residues in TM1, TM2, TM3, TM5, TM7, and helix 8 (Kapoor et al., 2017). Also, TM6 bending and intra-helical backbone hydrogen bond rearrangement were only observed with morphine- but not with TRV-130-bound µOR (Kapoor et al., 2017).

Mafi and co-workers compared morphine, DAMGO, and TRV-130 in MD simulations with the β-arrestin2-stabilized active phosphorylated µOR (Mafi et al., 2020). Accordingly, in the presence of non-biased agonists, β-arrestin2 coupled to the phosphorylated µOR by forming more polar connections with ICL2 and either the ICL3 or the cytoplasmic region of TM6. In contrast, TRV-130 induced a reposition of TM6 in the cytoplasmic region of the µOR by forming more polar interactions with TM2 and TM3. This repositioning hinders β-arrestin2 from properly binding to the phosphorylated µOR.

PZM21 was bound to µOR, TM5-6 and TM7 showed a larger outward and less inward movement, respectively (Figure 1C) (Liao et al., 2021). Also, the further outward movement of TM5–7 of the PZM21-bound µOR created a larger cavity potentially favorable for G protein binding. Zhao et al. analyzed and compared the flexibility of the loop region of PZM21 with morphine and TRV-130, and they found that the protein root mean square fluctuation (RMSF) values of morphine- and PZM21-bound µOR in the ECL1-3 and ICL3 regions were significantly smaller than those of TRV130-bound µOR (Zhao et al., 2020).

## Discussion and Conclusion

The introduction of the two active conformational structures of the µOR now allows a more precise analysis of ligand-specific changes in the receptor. Reviewing the increasing amount of the data regarding ligand-specific structural changes in µOR activation, certain tendencies can be observed (Table 1; Figure 1C). The current data proved that ligand recognition largely depends on the structural properties of the ligand. The highlighted ligands in this review differ in terms of flexibility, H-bond capabilities, and energy landscapes (Podlewska et al., 2020; Vo et al., 2020; Giannos et al., 2021). For instance, morphine and fentanyl despite being in contact with similar residues (Figure 1B), the binding pose itself is significantly different since morphine is more rigid and compact, while fentanyl is more flexible with an elongated shape. On the other hand, BU72 and DAMGO structurally differ significantly and there is also a difference regarding the depth of their binding pose. However, the conformation of the active binding pocket is highly similar. In the case of biased agonists TRV-130 and PZM21, it seems that they accomplish stronger and/or more contact with the receptor compared to unbiased ligands.

There are significantly more differences than similarities when it comes to forwarding the ligand-binding signal to the intracellular regions of the receptor. There are subtle, but important ligand-specific changes within the molecular switches; for instance, the different torsion angles or distances between the involved residues (Figure 1C). As mentioned above, such minor changes might also explain the higher efficacy of fentanyl (Ricarte et al., 2021) or β-arrestin coupling (de Waal et al., 2020). Another interesting finding is that with PZM21 the difference in rotations of certain residues can be associated with its lower bias effect (Zhao et al., 2020). Such delicate changes induce larger-scale movements for the µOR, which eventually dictate the faith of the receptor’s signaling pathway and possibly it’s degree of desensitization. These larger movements in essence allow a physical barrier or a favorable position for either the G-protein or β-arrestins, depending on the bound ligand.

In conclusion, ligand-specific µOR activation is defined by the following: 1) distinct number and/or degree of residue contacts within the ligand-binding pocket; 2) ligand-specific subtle changes within the residues (with respect to torsion angles and distances) of the TM regions, and as a consequence 3) triggers large-scale movements, toggles in certain domains of the receptor defining the type of downstream signaling of the µOR, as well as the degree of receptor desensitization. Further mapping these steps might open new strategies to develop opioid agonists with reduced analgesic tolerance and other side effects.
